# The practice of physicians and nurses in the Brazilian Family Health Programme – evidences of change in the delivery health care model

**DOI:** 10.1186/1478-4491-4-25

**Published:** 2006-11-15

**Authors:** Ellen M Peres, Ana M Andrade, Mario R Dal Poz, Nuno R Grande

**Affiliations:** 1 Faculty of Nursing, University of the State of Rio de Janeiro, Rio de Janeiro, Brazil; 2 Faculty of Dentistry, University of the State of Rio de Janeiro, Rio de Janeiro, Brazil; 3 Institute of Social Medicine, University of the State of Rio de Janeiro, Rio de Janeiro, Brazil and Tools, Evidence and Policy Unit at Human Resources for Health Department, World Health Organization, Geneva, Switzerland; 4 Emeritus Professor, Universidade do Porto, Porto, Portugal

## Abstract

The article analyzes the practice of physicians and nurses working on the Family Health Programme (Programa de Saúde da Família or PSF, in Portuguese).

A questionnaire was used to assess the evidences of assimilation of the new values and care principles proposed by the programme.

The results showed that a great number of professionals seem to have incorporated the practice of home visits, health education actions and planning of the teams' work agenda to their routine labour activities.

## Background

Brazilian health care systems have been subjected to many changes since 1988, when the new federal constitution (Brazilian major law) was promulgated. This Constitution instituted the National Health System – SUS ("Sistema Único de Saúde") – which was to be organized based upon the principles of universality, equity, continuous care through different levels, administrative decentralization and community participation in decision-making.

Over the last decade, a series of reforms have been undertaken, transferring responsibility for the administration and provision of public services in health to the level of the municipalities – one level below the states. In general, the management of the health system has improved with this transfer of federal resources and responsibility [1-3].

Funding levels have increased, allowing an expansion of services – the most significant being the Family Health Programme (Programa de Saúde da Família or PSF, in Portuguese) which has to date been implemented in 60% of the municipalities and which has been linked to the development of a cadre of community health workers known as Health Communitarian Agents [4]. Finally, more democratic mechanisms for community participation in decision making were established.

The Family Health Program (PSF), launched in 1995, is considered an innovative mechanism for extending access and promoting equity. The programme integrates public health actions with care and treatment and is based on many successful experiences as observed in other health systems, such as Canada, UK and Cuba [5,6].

One of the main reasons for the success of this model is that it emphasizes health promotion and preventive care, without forgetting treatment care, in a situation where providers perceive their role to be one of prevention as well as treatment. This simple shift in the perception of roles has been shown to be critical in predicting provider behaviour.

Each PSF team was constituted by one family physician, one nurse, two auxiliary nurses, and four to six community health agents and is responsible for a catchment area that includes 600–1000 families [7].

The PSF teams serve as the gateway to the health care services for individuals within the defined territory. In addition to direct assistance, the teams carry out a health situation analysis in collaboration with community leaders and organize their service in accordance with the population's specific health profile.

The PSF implementation brings about an important change in the health system: previously organized by service demands, it is now organized based on the supply of services, in a context of interdisciplinary and intersectorial interventions. As a result, humanization of health care and best client satisfaction are observed.

One of the critical problems faced by health authorities and health service managers is the lack of personnel trained to respond to the new demands of reoriented health care services. This includes not only family physicians and nurses to serve in primary health care programs, but also non-traditional members of the health team, such as counsellors, adult education specialists, anthropologists, community health promotion agents, etc.

To face these problems, the Brazilian Ministry of Health created the Training Centres In Family Health (TCFH) which are regionally distributed and aim to develop human resources competencies in family health [8].

This paper is one of the results of a project supported partially by the World Health Organization (WHO) [9] that aimed to evaluate the impact of the activities of Rio de Janeiro's Training Centre in Family Health (RJ-TCFH) upon the profile of human resources engaged in the Family Health Programme in some municipalities of the State of Rio de Janeiro [10].

Expected as an immediate consequence of the RJ-TCFH actions is a shift in health professionals' practices from individual medical care, early specialization, frequent and unnecessary use of high technology procedures and lack of humanization, to a model which emphasizes health promotion and preventive care – without forgoing curative care – working with a referenced population in a defined territorial basis. The teams should establish a link with the families, and base their work on a broad concept of health that makes use of interdisciplinary and intersectoral actions to improve the quality of the population's life.

Therefore, the selected indicators for this study refer to activities that should be incorporated into health professional practices in order to change the health care delivery model: home visits, health education activities, intersectoral actions, as well as participation in meetings with community leaders and in the Local Health Council. Some aspects of work organization, such as the planning of the team's actions and the use of local indicators for such planning, were also investigated.

Adhesion to those practices will indicate that the professional has changed his attitude, shifting from curative care based on a negative health concept to health promotion, founded in consistent educational activities and addressing the families of the catchment area.

## Methodology

The objective of the study was to explore and characterize the practice of the physicians and nurses of the Family Health Programme team, looking for evidence of the development of activities reflecting the values and principles proposed by the programme.

The six municipalities of the State of Rio de Janeiro where the Academic Institutions that compose the Rio de Janeiro's Training Centre in Family Health (TCFH) are situated were selected for this study.

A questionnaire was submitted to 209 professionals – 78 physicians and 131 nurses – working in Family Health Teams of the selected municipalities and who had previously participated in the Specific Training in Family Health provided by the RJ's TCFH and had accepted to contribute to the study.

The questionnaire aimed to gather information about the following.

a) Home visits:

- the number of home visits accomplished per month by the professional.

- the restriction of home visits to patients lying in bed or having locomotion disabilities (answering either 'yes' or 'no').

- facts that most frequently motivated the scheduling of home visits (options: the request by another PSF member; the PSF week plan; direct request by a community family).

b) Work planning by the Family Health Team:

- the main focuses of the last routine PSF meeting (options: decision-making about administrative issues; reading and discussing of scientific issues or debate of selected cases; elaboration of the PSF week plan; elaboration of the PSF month plan; communication of recent facts to the whole team).

- the use of local indicators for the planning and monitoring of PSF actions.

c) Health education activities:

- participation in health education activities as part of the work in the PSF ('yes' or 'no').

- the number of days per month dedicated by the professional to the accomplishment of health education activities.

d) Compromise towards the assisted community

- participation in community meetings as part of the work in the PSF ('yes' or 'no').

- the number of days per month dedicated by the professional to community meetings.

- participation in the Local Health Council of the PSF area.

- the accomplishment of actions involving different public sectors (e.g. education, sanitation, security, etc) as part of the work in the PSF.

## Results and Discussion

The home visit (HV) is reserved for health promotion as well as for health surveillance practices. This is a new approach, moving away from merely fragmented activities (e.g. communicable diseases control). In the PSF, the home visit is one of the core actions to improve the situational analysis of the designated population, looking at the social dynamics of that specific community. Therefore, there is an enlargement of the objectives, which include health promotion, prevention, treatment and rehabilitation in a perspective of an integral and humanized care.

The HV is here a central subject for the analysis. There is a consensus that the health professionals do not have opportunities in their training to develop the necessary competences for the home visit practice and therefore do not consider it a priority or even part of primary health care. The home visit, therefore, represents an indicative of change of the professionals' practice and their adhesion to the principles and guidelines of PSF.

**Figure 1:**
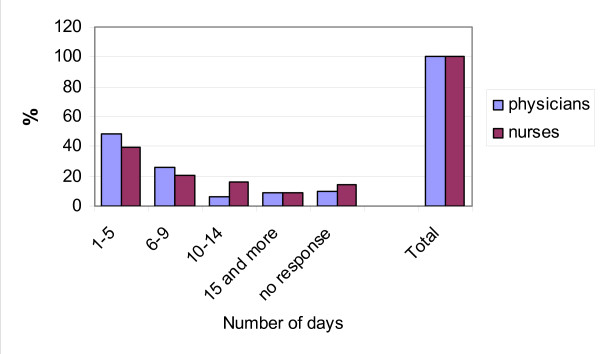
Number of days a month including home visits.

Figure 1 shows the number of days per month in which the professionals perform home visits. The average value was 7.1 days for the physicians and 8.1 for the nurses (also interpretable as twice a week). All the professionals claimed to perform home visits. However, the high percentage (10.3% of physicians and 14.5% of nurses) of lack of answer to the question concerning the number of days per month suggests that there may be a number of them who do not want to admit their failure to perform home visits.

When asked about the circumstances that lead the professionals to perform home visits (Table 1), the number of answers stating that home visits were restricted to patients lying in bed or having locomotion disability was somewhat high (30.7% of physicians and 20.6% of nurses), although the majority of professionals claimed that they did not make this restriction. As could be expected, the former group also performed a lower number of home visits per month (Table 2).

**Table 1:** Reasons for home visits

**Reasons**	**Physicians**		**Nurses**	
	
	**n**	**%**	**n**	**%**
	
	46	59.0	84	64.1
"The visits are only carried out to bed-bound patients or that cannot walk"	24	30.7	27	20.6
no response	8	10.3	20	15.3

**Total**	**78**	**100.0**	**131**	**100.0**

**Table 2:** Average number of home visits monthly, by their reasons

**Reasons**	**Physicians**	**Nurses**
	
	**average**	**average**
"The visits are carried out not only for bed-bound patients or those who cannot walk"	7.7	8.6
"The visits are only carried out to bed-bound patients or those that cannot walk"	5.8	6.4

In relation to the criteria used for the scheduling of home visits, most professionals – 69.2% of physicians and 61.1% of nurses (Table 3) – said that the scheduling is determined by the Family Health Team week plan, which is the expected attitude of a Family Health Team working from an interdisciplinary point of view. As shown in Table 4, this criterion was also the one that corresponded to the higher averages of home visits by month, although the difference to the averages relative to the other criteria is not great.

**Table 3:** Most frequent criteria to arrange a home visit

**Criteria**	**Physicians**		**Nurses**	
	
	**n**	**%**	**n**	**%**
The appointment is fixed by the team's weekly planning.	54	69.2	80	61.1
The appointment is fixed when requested by a team member.	27	34.6	47	35.9
The appointment is fixed when requested by a community family.	20	25.6	27	20.6
Other	4	5.1	6	4.6
No response	1	1.3	1	0.8

**Table 4:** Average number of home visits, by the most frequent criteria adopted

**Criteria**	**Physicians**	**Nurses**
The appointment is fixed by the team's weekly planning	6.8	8.1
The appointment is fixed when requested by a team member	6.4	7.2
The appointment is fixed when requested by a community family	5.9	7.3
Other	5.8	7.4

The results indicate that the home visit is being incorporated into the professionals' practice. However, it is necessary to rethink the real objectives of the home visits to those populations. For instance, should the home visits undertaken by graduate professionals be planned for families at risk? Should HVs prioritize the families to which attention cannot be given in the health centre? Is it possible, for those professionals, to perform actions of health surveillance for all of the designated families, considering the other responsibilities they have in the health centre? Clearly, if the resource constraints do not allow for a change in the relationship between the team and the population, the expectations related to the home visits should be reviewed.

As we can tell from Table 5, 73% of physicians and 61.2% of nurses claimed to perform health education activities from 1 to 5 days per month, with an average of 4.4 and 5.6 days respectively, i.e. an average of once a week. Thus, health education activities also seem to have been incorporated into the practices of physicians and nurses working in family health teams, constituting an important strategy for the reinforcement of health promotion.

**Table 5:** Number of day monthly with activities on health education

**Number of days**	**Physicians**		**Nurses**	
	
	**n**	**%**	**n**	**%**
Zero days	1	1.3	2	1.5
From 1 to 5 days	57	73.0	81	61.8
From 6 to 9 days	7	9.0	20	15.3
From 10 to 14 days	4	5.1	3	2.3
More than 15 days	2	2.6	11	8.4
No response	7	9.0	14	10.7

**Total**	**78**	**100.0**	**131**	**100.0**

The community meetings are dedicated to social participation in defining priorities and adopting strategies to address problems.

The number of days per year that the professionals participate in meetings with community members showed great variation (Table 6), with an average of 4 days. This average can be considered somewhat low: for an adequate integration of the health team and the community, it is expected that such meetings should occur at least once a month, which would result in an average of at least 10 meetings per year.

**Table 6:** Number of days annually to attend community meetings

**Number of days**	**Physicians**		**Nurses**	
	
	**n**	**%**	**n**	**%**
Zero days	30	38.5	47	35.9
From 1 to 5 days	17	21.8	43	32.8
From 6 to 9 days	5	6.4	7	5.3
From 10 to 14 days	10	12.8	14	10.7
More than 15 days	3	3.8	6	4.6
No response	13	16.7	14	10.7

**Total**	**78**	**100.0**	**131**	**100.0**

The assessment of the participation in the Local Health Council of the HFT (Table 7) showed that only 15.3% of the professionals gave a positive answer to this question, and only 4.3% said that their participation was frequent; 6.2% participated occasionally and 4.8% rarely. Of all the professionals, 43.1% admitted they had never participated and 38.3 said that there was not any Local Health Council in their PSF area. These results indicate that this activity, important for social control, is not yet recognized by the professionals as part of their tasks in the family health programme.

**Table 7:** Degree of participation in the Local Health Council relating to the Family Health Team

**Degree of participation**	**n**	**%**
Frequently	9	3.0
Sometimes	16	5.4
Almost never	12	4.0
Never	132	44.3
Local Health Council does not exist	120	40.3
No response	9	3.0

**Total**	**298**	**100.0**

The final assessed activity was the participation in intersectoral actions. Table 8 shows that only half of the professionals have already participated in these kind of actions. This means that the other half still has the view that the health professional's obligations are restricted to what is considered the "health area", forgetting that health, in a broader concept, is influenced by a large spectrum of influences of variable kinds – living conditions, habits, social conditions, environment conditions and so on – and that health professionals have the responsibility of interacting with these other sectors in order to provide health promotion to the population they attend.

**Table 8:** Previous participation in intersectoral activities.

**Participation**	**n**	**%**
Never participated	144	48.3
Already participated	134	45.0
No response	20	6.7

**Total**	**298**	**100.0**

On the other hand, the overload of work concerning the population under the responsibility of a team can be a barrier to the development of other actions to complement health care. One can even question whether the intersectoral actions should not be part of the local government activities towards healthy communities, reducing the current burden and responsibility of the Family Health Team.

The study also investigated some aspects of work organization: the planning of the team's actions and the use of local indicators for such planning.

When asked about the main focuses of the last routine meeting of the PSF, 57% of the professionals said that it had been the elaboration of the PSF week plan (Table 9), indicating that more than half of the health teams have incorporated the weekly planning of their activities into their practices. Nevertheless, only 52.7% of the professionals (Table 10) claimed to use local indicators frequently for the planning and monitoring of their team's actions. Thus, this is a point that should be reinforced by educative interventions to be provided by the RJ'TCFH: the importance of local indicators to guide the team's actions and of selecting the most adequate indicators to each individual (team) reality.

**Table 9:** Focus of the last team meetings

**Principal focus**	**n**	**%**
Planning of the next week's activities	118	57.0
Sharing information on activities already completed	73	35.3
Learning of relevant issues/discussion of clinical cases	69	33.3
Decisions on administrative procedures	56	27.1
Planning of the next month's activities	53	25.6
Other	6	2.9
No response	2	1.0

**Table 10:** Frequency on the use of local level indicators for planning and monitoring

**Frequency**	**n**	**%**
Frequently	158	52.9
From time to time	97	32.6
Almost never	19	6.4
Never	13	4.4
No response	11	3.7

**Total**	**298**	**100.0**

## Conclusion

The practice of physicians and nurses of the PSF teams included in this research showed their assimilation of some of the new values and care principles proposed by the PSF that can result in a change to the health care delivery model [11,12].

The accomplishment of home visits not restricted to patients in bed and of health educations actions point to a paradigm shift towards the integration of health promotion, preventive care and treatment and to the humanization of health care. The previous planning of the teams' work, undertaken by the whole team, indicates a shift towards interdisciplinary work. The use of local indicators for such planning shows that the professionals are now working with a referenced population in a defined territorial basis.

The results suggest that resource constraints, leading to a low proportion of health care workers by designated population, make more difficult the adoption of the desired practices by the professionals, especially those related to intersectoral and local community actions.

Although the change in the professionals' practices has not yet reached 100% of physicians and nurses working in the PSF teams, we can presume that this is an on-going process of changes and that other actions, such as continuous education, can contribute to strengthening the programme.

## Competing interests

The principal author (EMP) was coordinator of the Centre of Training, Education and Continuous Education on Family Health in the State of Rio de Janeiro, Brazil from 1999 to 2002.

## Authors' contributions

All authors contributed equally to the design, analysis and writing of the paper.
